# The Role of the *Salmonella spvB* IncF Plasmid and Its Resident Entry Exclusion Gene *traS* on Plasmid Exclusion

**DOI:** 10.3389/fmicb.2020.00949

**Published:** 2020-05-15

**Authors:** Mopelola Oluwadare, Margie D. Lee, Christopher J. Grim, Erin K. Lipp, Ying Cheng, John J. Maurer

**Affiliations:** ^1^Department of Population Health, University of Georgia, Athens, GA, United States; ^2^Department of Biomedical Sciences and Pathobiology, Virginia Polytechnic Institute and State University, Blacksburg, VA, United States; ^3^Center for Food Safety and Applied Nutrition, U.S. Food and Drug Administration, Laurel, MD, United States; ^4^Department of Environmental Health Science, University of Georgia, Athens, GA, United States; ^5^Department of Animal and Poultry Sciences, Virginia Polytechnic Institute and State University, Blacksburg, VA, United States

**Keywords:** *Salmonella*, virulence plasmid, antibiotic resistance, entry exclusion, conjugation

## Abstract

*Salmonella enterica* cause significant illnesses worldwide. There has been a marked increase in resistance to fluoroquinolones and β-lactams/cephalosporins, antibiotics commonly used to treat salmonellosis. However, *S. enterica* serovars vary in their resistance to these and other antibiotics. The systemic virulence of some *Salmonella* serovars is due to a low copy number, IncF plasmid (65–100 kb) that contains the ADP-ribosylating toxin, SpvB. This virulence plasmid is present in only nine *Salmonella* serovars. It is possible that the *spvB*-virulence plasmid excludes other plasmids and may explain why antibiotic resistance is slow to develop in certain *Salmonella* serovars such as *S*. Enteritidis. The distribution of plasmid entry exclusion genes *traS*/*traT* and *traY*/*excA* are variable in *Salmonella* IncF and IncI plasmids, respectively and may account for differences in emergent antimicrobial resistance for some *Salmonella* serovars. The goal of this study is to determine the contribution of the *Salmonella spvB*-virulence plasmid in F-plasmid exclusion. From conjugation experiments, *S*. Typhimurium exhibited lower conjugation frequency with incFI and incFII plasmids when the *spvB*-virulence plasmid is present. Furthermore, introduction of cloned incFI *traS* into a “plasmidless” *S*. Typhimurium LT2 strain and *Escherichia coli* DH5α excluded incFI plasmid. However, deletion of the virulence plasmid *traS* did not affect plasmid exclusion significantly compared to a *spvB* control deletion. In addition, differences in F plasmid conjugation in natural *Salmonella* isolates did not correlate with IncF or SpvB-virulence plasmid genotype. There appear to be other plasmid or chromosomal genes at play in plasmid exclusion that may be responsible for the slow development of antibiotic resistance in certain serovars.

## Introduction

*Salmonella enterica* is responsible for 78 million illnesses and 59 thousand deaths per year, worldwide (Havelaar et al., 2015). Non-typhoid *Salmonella* are primarily transmitted via fecal contamination of meat, eggs, dairy products, fruits, nuts, and vegetables (De Buyser et al., 2001; Brar and Danyluk, 2018; De Cesare, 2018; Li et al., 2018; Omer et al., 2018). In the United States, *S. enterica* causes 1 million illnesses each year, resulting in 19 thousand hospitalizations (Scallan et al., 2011). Although most *Salmonella* infections are largely treatable with antibiotics, a disturbing trend is the rise of multidrug resistant (MDR) *Salmonella* (Wasyl et al., 2015; Iwamoto et al., 2017; Tyson et al., 2017; Duong et al., 2018), especially to fluoroquinolones, β-lactams, and cephalosporins; antibiotics frequently used to treat these infections (Collard et al., 2007; Tribble, 2017; Duong et al., 2018). According to the National Antimicrobial Resistance Monitoring System (NARMS) 2015 Annual Report, 12.4% of *S. enterica* isolates tested were resistant to three or more classes of antibiotics [Centers for Disease Control and Prevention (CDC), 2016]. However, *Salmonella* serovars vary substantially in their susceptibility to antimicrobials tested in the NARMS panel. For example, 77.7% of *S*. Enteritidis isolates are pan-susceptible compared to just 28.3% of *S*. Typhi isolates [Centers for Disease Control and Prevention (CDC), 2016].

Mobile genetic elements, such as plasmids and transposons, are responsible for the transmission and subsequent dissemination of antimicrobial resistance (Partridge et al., 2018). However, a few *Salmonella* serovars such as Enteritidis are slow to develop antimicrobial resistance despite their presence in environments rich in antimicrobial resistance genes (Liljebjelke et al., 2017). The question that arises is why are some *Salmonella* serovars slow to develop antibiotic resistance compared to others?

Plasmids are often important vehicles for disseminating antibiotic resistance, however some *Salmonella* serovars are slower in developing antibiotic resistance compared to others. A significant genetic barrier to plasmid transmission and therefore development of antimicrobial resistance may be the resident *spvB*-virulence plasmid. Like virulence plasmids in *E. coli* pathovars (Johnson and Nolan, 2009), the *Salmonella* virulence plasmid belongs to IncF incompatibility group, and specifically contains the FIC and FII replicons present in F and R100 plasmids, respectively (Villa et al., 2010). Similarly, newer β-lactam/cephalosporin and quinolone resistance genes reside on IncF plasmids. Those same resistance genes in *Salmonella*, however, reside on plasmid incompatibility groups other than IncF (Carattoli, 2009).

Some *Salmonella* serovars contain a large molecular weight plasmid that enables it to proliferate in the reticuloendothelial system and cause systemic infection (Gulig and Curtiss, 1987). The key virulence factor SpvB is an ADP-ribosylating toxin that interferes with phagocyte function in the host (Lesnick et al., 2001). This plasmid is present in the non-typhoid *Salmonella* serotypes Abortusovis, Choleraesuis, Derby, Dublin, Enteritidis, Gallinarum/Pullorum, Paratyphi C, and Typhimurium (Boyd and Hartl, 1998; Feng et al., 2012). While the *spv* operon provides a small, but crucial growth advantage in bovine and human monocyte-derived macrophages (Libby et al., 2000), it does not appear to be essential to *Salmonella*'s ability to cause gastroenteritis (Horiuchi et al., 1991). Other virulence loci present on the virulence plasmid are: the *pef* genes (plasmid-encoded fimbriae) which mediate adhesion to the small intestine and contributes to fluid accumulation in infant mouse model (Baumler et al., 1996); *srgA*, SdiA-regulated genes and putative disulphide bond oxidoreductase (Feng et al., 2012); *mig-5*, a macrophage-inducible gene and putative carbonic anhydrase (Feng et al., 2012); and *rck*, which encodes a 17-KD outer membrane protein that confers complement resistance (Vandenbosch et al., 1989).

The *spvB-v*irulence plasmid is a conjugative, IncF plasmid (Ahmer et al., 1999; Villa et al., 2010). The F-plasmid, or “Fertility” (F) factor, was the earliest of self-transmissible plasmids studied, and it has served as a model for understanding plasmid replication, partitioning, maintenance, and transfer. This plasmid contains 40 genes clustered together in a 33.3 kb transfer region (*tra*) that mediates physical transfer of plasmids between bacterial cells. The F plasmid transfer begins when the F pilus makes contact with one or more recipient cells, which leads to the formation of a mating pair aggregate (Frost et al., 1994). The F pilus retracts to bring donor and recipient adhesion sites into contact to form a transfer pore. The OmpA protein along with TraG and TraN stabilize the pore, which leads to the stabilization of the mating pair aggregates (Klimke and Frost, 1998; Anthony et al., 1999). Over time, the mating pair stabilize and they will not disassociate, even with the addition of sodium dodecyl sulfate (SDS) (Achtman et al., 1978). There are two main genes reported to be involved in mating pair stabilization: *traN* in the outer-membrane and *traG* in the inner-membrane (Manning et al., 1981; Klimke and Frost, 1998).

Plasmids are similar to bacteriophages in that they are “selfish DNA” that contain attributes that favors their spread and retention within their bacterial host, while excluding similar competing DNA molecules. The distribution of plasmid incompatibility groups, within a bacterial population or microbial community, affects plasmid transmission. Competition for initiation of DNA replication or partitioning between the dividing daughter cells affects the plasmid's persistence within a community of like plasmids (Novick, 1987; Bouet et al., 2007). Plasmids with similar replicons and partitioning apparatus are incompatible. This incompatibility is often tied to exclusion, mechanisms that limit plasmid transfer by disrupting mating aggregates (surface exclusion) or inhibiting DNA transfer in the presence of mating aggregates (entry exclusion) (Garcillan-Barcia and de la Cruz, 2008).

Like lysogenic bacteriophages, plasmids have several mechanisms for preventing “super infection” of its bacterial host with similar plasmids. Plasmid incompatibility, the inability of similar plasmids to coexist in the same bacterial cell, involves either interference with initiation of plasmid replication (Novick, 1987) or partitioning of competing plasmids between daughter cells (Bouet et al., 2007). Another mechanism, exclusion involves interference with plasmid transfer of those belonging to the same incompatibility group. For plasmids belonging to *IncF* incompatibility group, *traS* and *traT* are primarily responsible for this exclusion (Garcillan-Barcia and de la Cruz, 2008). TraT is an outer membrane lipoprotein that blocks conjugation by preventing the formation of stable mating aggregates (Sukupolvi and O'Connor, 1990). TraS, localized in the inner membrane, functions to inhibit DNA transfer even after the formation of stable mating aggregates by recognizing its cognate TraG in the donor cell (Achtman et al., 1979; Firth and Skurray, 1992).

**Figure 1:**
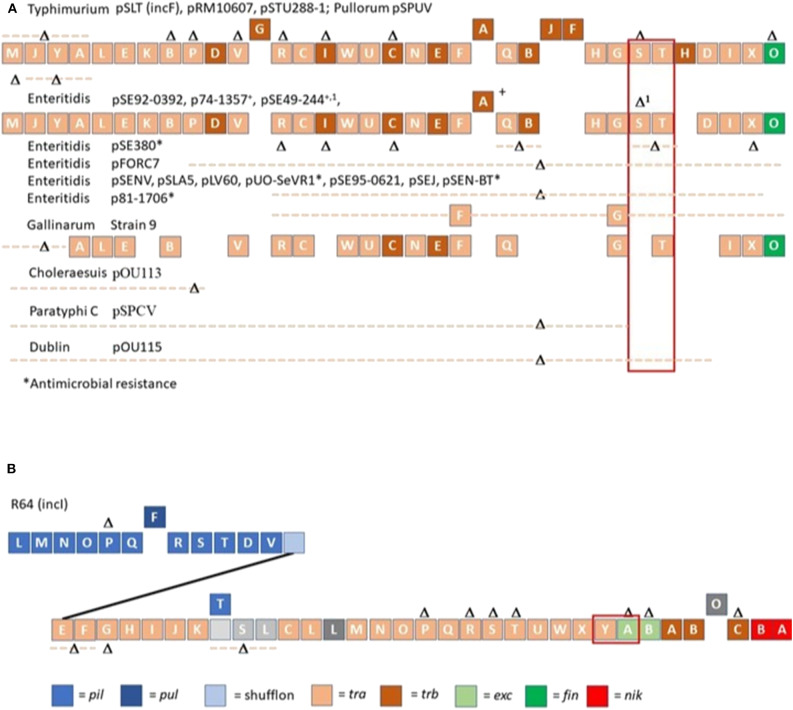
Organization of conjugation genes associated with IncF **(A)** and IncI **(B)** plasmids in *Salmonella* isolated from non-domestic animals and water. R64 (IncI) and *Salmonella* Typhimurium LT2 *spvB*-virulence plasmid (pSLT; IncF) served as templates for comparing gene organization of *Salmonella* plasmids. Different colors were used to depict the different classes of conjugation genes. Gene (s) or contiguous plasmid segment missing in *Salmonella* isolates are denoted as Δ or Δ with dotted line. A solid line was used to connect *pil* with *tra* operon in R64, IncI plasmid. Blocks above plasmid backbone for R64 or pSLT denotes gene(s) only present in other *Salmonella* plasmids. The exclusion genes *traS, traT* and *traY, excA* are bracketed in red.

Genomic comparisons of *spvB*-virulence plasmids in *S*. Choleraesuis, *S*. Dublin, and *S*. Enteritidis, using *S*. Typhimurium LT2 pSLT as the prototype, self-transmissible virulence plasmid, revealed large deletions within the *tra* operon, including *traS* responsible for entry exclusion (Yu et al., 2006; Hong et al., 2008). The *S*. Choleraesuis, *S*. Dublin and *S*. Enteritidis virulence plasmids are neither mobilizable nor self-transmissible due to the absence of *oriT* and most *tra* genes (Yu et al., 2006; Hong et al., 2008). As more *Salmonella* virulence plasmids have been sequenced, there is significant differences with regards to *tra* gene(s) deletion(s); where most deletions include both entry exclusion genes *traS* and *traT* or *traS* alone (Figure 1A).

The SpvB virulence plasmid gene, *traS* is an important genetic barrier to development of plasmid-mediated antimicrobial resistance. If this is the case, natural *Salmonella* isolates possessing the SpvB virulence plasmid are less likely to acquire other large molecular weight plasmids including those that mediate plasmid-mediated antibiotic resistance. The purpose of this study is to determine whether the *Salmonella* SpvB-virulence plasmid, and specifically *traS*, can actively exclude other incF plasmids from entering the cell.

## Materials and Methods

### Bacterial Strains and Plasmids

Table 1 describes bacterial strains and plasmids used in this study. *Escherichia coli* strains XK1200 and MC4100 served as bacterial hosts for IncFI and IncFII plasmids pOX38-km and R100, respectively. Bacterial cultures were grown overnight at 37°C in Luria-Bertani (LB) medium with appropriate antibiotic at the following concentrations: tetracycline (10 μg/ml), kanamycin (30 μg/ml), nalidixic acid (64 μg/ml), rifampicin (64 μg/ml), and chloramphenicol (64 μg/ml). *Salmonella* Typhimurium pSLT^−^ strain was constructed by transducing pStLT203 Ω *parA*::Km (Tinge and Curtiss, 1990) into *S*. Typhimurium strain LT2 using P22 HT *int* (Provence and Curtiss, 1994). A kanamycin-resistant, *S*. Typhimurium transductant was passaged in LB without antibiotics and subsequently screened for sensitivity to kanamycin (Tinge and Curtiss, 1990). Loss of pSLT virulence plasmid was confirmed by polymerase chain reaction (PCR) analysis (Swamy et al., 1996). Defined deletions in *traS* or *spvB* were introduced into *S*. Typhimurium virulence plasmid using λ *red* recombineering approach described by Datsenko and Wanner (2000). Table 2 describes PCR primers and conditions used to construct λ *red* knockouts. *Escherichia coli* and *S*. Typhimurium LT2 strains were transformed using electroporation protocol described by Dower et al. (1988).

**Table 1:** Bacterial strains and plasmids.

**Bacterial strain or plasmid**	**Description^a^^,^^b^**	**References**
***E. coli***		
XK1200	Nal^r^; F^−^*lacΔU124* Δ(*nadA aroG gal attλ bio*) *gyrA*	Anthony et al., 1996
MC4100	Sm^r^; F^−^*araD139 Δ*(*argF*-*lacU169*) *rpsL150 relA1 flbB3501 deoC1 ptsF25 rbsR*	Anthony et al., 1996
DH5α	Nal^r^; F^−^*supE44 ΔlacU169* (ϕ80 *lacZΔM15*) *hsdR17, recA1 endA1 gyrA96 thi-1 relA1*	Hanahan, 1983
***S. enterica***		
LT2	*S*. Typhimurium; *spvB*-virulence plasmid^+^ (pSLT; IncFII)	Sanderson and Roth, 1988
LT2R	*S*. Typhimurium LT2; Rif^r^, *spvB*-virulence plasmid^+^ (pSLT)	This Study
pSLT^−^	*S*. Typhimurium LT2; Rif^r^, *spvB*-virulence plasmid^−^	This Study
pSLT^−^*traS^+^*	*S*. Typhimurium pSLT^−^; Rif^r^, Tc^r^, pRS31 (FI *traS^+^*)	This Study
LT2R Δ*traS*^c^	*S*. Typhimurium LT2R; Rif^r^, *ΔtraS*, pSLT^+^	This Study
LT2R Δ*spvB*^c^	*S*. Typhimurium LT2R; Rif^r^, *ΔspvB*, pSLT^+^	This Study
98A-33516R	*S*. Typhimurium 98A-33516; Rif^r^, *spvB*-virulence plasmid^−^; Songbird isolate	Hudson et al., 2000
98A-28238R	*S*. Typhimurium 98A-28238; Rif^r^, *spvB*-virulence plasmid^+^, pSLT *traS*^−^; Songbird isolate	Hudson et al., 2000
108709R	*S*. Choleraesuis 108709; Rif^r^, *spvB*-virulence plasmid^+^	This Study
564R	*S*. Dublin 564; Rif^r^, *spvB*-virulence plasmid^+^, pSLT *traS*^−^; Cattle isolate	This Study
**Plasmids**		
pOX38-Km	IncFI plasmid; Km^r^, Tra^+^ Conjugative	Anthony et al., 1999
pRS31	FI *traS*^+^ in pSC101; Tc^r^	Anthony et al., 1999
R100-1	IncFII plasmid; Cm^r^ Fa^r^ Sm^r^ Sp^r^ Su^r^ Tc^r^, Tra^+^ conjugative	Anthony et al., 1999
pKD3	Template plasmid for *cat* cassette used in recombineering λ red mediated insertions and subsequent “flippase” mediated excisions/deletions; Ap^r^, Cm^r^	Datsenko and Wanner, 2000
pKD20	*repA101ts*, λ *γ, β, exo*; Ap^r^	Datsenko and Wanner, 2000
pCP20	Temperature-sensitive replicon and inducible “flippase” (Flp) for deleting *cat* and adjacent sequences to create targeted deletions; Ap^r^, Cm^r^	Cherepanov and Wackernagel, 1995

a *Ap, ampicillin; Cm, chloramphenicol; Fa, fusaric acid; Nal, nalidixic; Rif, rifampicin; Sp, spectinomycin; Sm, streptomycin; Su, sulfonamide; Tc, tetracycline*.

b *Clinical isolates were identified as positive or negative for spvC (Swamy et al., 1996) or pSLT traS*.

c *aph cassette was removed with pCP20 (Cherepanov and Wackernagel, 1995; Datsenko and Wanner, 2000)*.

**Table 2:** PCR primers.

**Target**	**Sequence**	**Expected size (bp)**	**Annealing temperature (°C)**	**References**
*spvB*	F:TCATACTCCAGCAGCAGACG	587	50°C	This Study
	R:AGCAGTTTTTATCGCCTGGA			
*spvB*-*aph* ins^a^	F:GTATCAGGATAAGCACAAACAGTAAGGCGATATCCG	1,100	57°C	This Study
	R:TCATCCAATTACCTTTATTTACCAACCATAGTTTTCTTATTA			
*spvC*	F:CGGAAATACCATCTACAAATA	669	40°C	Swamy et al., 1996
	R:CCCAAACCCATACTTACTCTG			
pSLT *traS*	F:ACCTGTCATTATTATCCTGC	400	55°C	This Study
	R:ATTATCCTGTTATTTGTCCTGC			
*traS*-*aph* ins^a^	F:CAGGAGATAGTGTATGTTGATACTAAATGGTTTTTCATCT	1,100	54°C	This Study
	R:TATCGCCATATTATTAGATATAAATTCTCAG			
FI *traS*	F:TCTGCCGGAAGAATTCCTAA	152	50°C	This Study
	R:CCGTCACTAAAATTGCACCA			

a *Primers used to create λ red—targeted knockouts with aph gene cassette (Datsenko and Wanner, 2000)*.

### Mating Assay

Conjugations were performed as follows. Bacteria grew as standing overnight cultures at 37°C in Luria-Bertani (LB) broth. The mating mix consisted of overnight cultures of donor strain (5 μl) and recipient strain (50 μl), in 5 ml of 10 mM MgSO_4_. Cells were collected on a 0.45 μm pore size cellulose filter membrane (Millipore Sigma; St. Louis, MO), and the filter was aseptically placed on Luria-Bertani (LB) plate containing 0.2% glucose and 10 mM MgSO_4_, cell side up. After overnight incubation at 37°C, a cell suspension was made by aseptically placing the filter in 5 ml of 10 mM MgSO_4_ and vortexing. The cell suspension was diluted 10-fold and plated on LB plates containing the appropriate antibiotic for selecting recipients or transconjugants. The conjugation frequency was determined from the number of transconjugants divided by recipients; averaging the results of duplicate matings, for three separate trials.

### PCR

Web-based software analysis program, Primer3Plus (https://primer3plus.com/cgi-bin/dev/primer3plus.cgi) was used to design primers, targeting *Salmonella* Typhimurium LT2 virulence plasmid genes *spvB* and *traS* genes. See Table 2 for description of primer sequences, PCR conditions, and expected size for PCR amplicons. The University of Georgia Molecular Instrumentation Laboratory synthesized the PCR primers. Genomic DNA was prepared as described by Sambrook et al. (1989). The PCR reaction mix contained 2 mM MgCl_2_, 0.1 mM primer, 0.2 mM nucleotide and 0.5-unit Taq DNA polymerase (Roche Molecular Biochemicals; Indianapolis, IN). PCR screens were performed using the Rapidcycler hot-air thermocycler (Idaho Technology; Salt Lake City, UT) with denaturation set at 93°C for 1 min; annealing as described in Table 2 for each primer set for 1 s, and primer extension at 72°C for 15 s for 30 cycles. Probes, for DNA: DNA hybridization, were prepared by PCR, substituting standard nucleotides with digoxigenin-labeled nucleotides (Roche Molecular Biochemicals) in the PCR reaction mix.

### Plasmid Extraction

Bacterial isolates were streaked from frozen glycerol stocks onto tryptic soy agar (Fisher Scientific) and plates were incubated overnight at 37°C. A bacterial suspension was made by inoculating Superbroth (Provence and Curtiss, 1994) (6 ml) with a single colony. Bacteria grew overnight at 37°C with aeration (235 rpm). Plasmid DNA was extracted from overnight cultures using the FosmidMAX DNA Purification Kit (Epicenter; Grand Island, NY). DNA samples were stored at −20°C. Gel electrophoresis separated plasmid DNA on a 0.5% agarose gel; at 44 V in E buffer (40 mM Tris-acetate, 2 mM sodium EDTA) for 16.5 h (88). Gels were stained with 1 X Sybr Gold (Invitrogen) in 1X TAE (40 mM Tris-acetate, 1 mM EDTA; pH 8.0) buffer while shaking for 30 min at 40 rpm. Gel images, illuminated on UV transilluminator, were captured with Molecular Imager Gel Doc XR System digital camera (BioRad; Hercules, CA).

### DNA: DNA Hybridization

Agarose gels were stored at 4°C before DNA transfer to nylon membranes. Gels were treated with HCl, followed with NaOH treatment (Sambrook et al., 1989), before the single stranded DNA was transferred to a nylon membrane using BioRad vacuum blotter. Single stranded DNA was UV-cross linked onto the nylon membranes. Membranes were covered in aluminum foil and stored at −80°C. DNA:DNA hybridization was performed as described by Sambrook et al. (1989) with hybridization and washes performed at 68°C. Bound probe was visualized, on nylon membranes, with anti-digoxigenin alkaline phosphatase conjugate and the nitroblue tetrazolium/5-bromo-4-chloro-3-indolylphosphate substrate as described by the manufacturer (Roche Molecular Biochemicals). The DNA probe generated using FI *traS* primers is specific for F-plasmid and its F-plasmid derivative pOX38. There is only 47% identity at the nucleotide level between the *traS* of pSLT and F-plasmid.

### Sequencing of *Salmonella* Genomes

*Salmonella enterica* strains were sub-cultured from frozen stocks onto Tryptic Soy Agar (TSA) (Fisher Scientific) plates amended with 5% sheep blood and incubated overnight at 37°C. Single isolated colonies were inoculated into Tryptic Soy Broth (TSB) (Fisher Scientific) and incubated overnight at 37°C, with shaking. Cell pellets were harvested by centrifugation at 6,010 × g for 5 min, and genomic DNA was extracted with the QIAcube automated sample preparation platform, using the QIAamp DNA mini protocol (Qiagen, Valencia, CA, USA).

Extracted genomic DNA was quantified using a Qubit 2.0 Fluorometer (Invitrogen- ThermoFisher, Waltham, MA, USA). Genomic DNA was diluted with nuclease-free water and sequencing libraries were prepared using the Nextera XT DNA Library Prep kit (Illumina, San Diego, CA, USA). Whole genome sequencing was performed on the MiSeq benchtop sequencer (Illumina, San Diego, CA, USA), utilizing 2 × 250 bp paired-end V2 chemistry. Raw sequence reads were deposited in the Sequence Read Archive (SRA) at NCBI. Fastq datasets were trimmed for quality, ambiguities (*n* = 0), and length (l > 150 bp), and then *de novo* assembled with CLC Genomics Workbench version 9.0 (CLC bio, Aarhus, Denmark). The draft genome sequence assemblies were annotated using Rapid Annotation using Subsystem Technology (RAST) (Aziz et al., 2008). The *Salmonella* genomes were searched for contigs containing genes annotated as “*tra*,” including *traI*. Plasmid incompatibility group was identified based on homology to the conjugative relaxase TraI of published, reference plasmid genomes for the F plasmid (IncF) (NCBI GenBank AP001918.1), R100 (IncF) (NCBI GenBank NC_002134.1), and R64 (IncI) (NCBI GenBank AP005147) (Fernandez-Lopez et al., 2017). Plasmid genome comparisons were limited to those belonging either to IncF or IncI, as these are the most studied conjugative plasmids, especially in terms of understanding entry exclusion (Furuya and Komano, 1994; Garcillan-Barcia and de la Cruz, 2008).

### Statistical Analysis

Chi-squared; and paired and unpaired Student tests were used to determine whether differences observed were significant.

## Results

### Distribution of Conjugative Plasmids in *Salmonella* Isolated From Non-domestic Animals and Water

Whole genome sequencing was performed on 161 *Salmonella* isolated from various animal species (reptiles, opossum, racoon, songbirds) and water in order to identify major conjugative plasmids. These isolates were chosen from sources that are not likely to be exposed to antibiotics; and their resident plasmids are more likely to reflect their natural state prior to antibiotic selection pressure. Approximately half of *Salmonella* isolates (*n* =161) possessed IncI (34.2%) or IncF plasmids (18.0%) (Figure 1B, Table 3). None of these plasmids possessed genes associated with antimicrobial or heavy metal resistance. *Salmonella* IncI and incF plasmids possessed plasmid exclusion genes *traY*/*excA* and *traS,T*, respectively. However, of the isolates that possessed one of these two plasmid types, only 10.9 and 13.8% had both plasmid exclusion genes *traY/excA* or *traS,T*, respectively (Table 3). Of *Salmonella* IncF plasmids (*n* = 29) identified, five possessed *spvB* (17.9%); the signature gene of *Salmonella* virulence plasmids. These *spvB* virulence plasmids, as well as the other IncF plasmids, were variable in the distribution of plasmid exclusion gene *traS* (Table 3).

**Table 3:** Distribution of conjugative IncI and IncF plasmids in *Salmonella* isolated from non-domestic animals and water.

**Plasmid^a^**	**Prevalence (*n* = 161)**	***spvB* (*n* =29^b^)**	**Plasmid exclusion genes**			
			***traY* (*n* = 55^c^)**	***excA* (*n* = 55^c^)**	***traS* (*n* =29^b^)**	***traT* (*n* =29^b^)**
*IncI*	34.2 %		100.0%	10.9%		
*IncF*	18.0%	17.2%			13.8%	100.0%
*IncI* or *IncF*	49.7%					
*IncI* and *IncF*	5.0%					

a *Plasmid type based on organization of R64 (IncI) or pSLT (IncF) tra locus*.

b *Total IncF plasmids*.

c *Total IncI plasmids*.

### Plasmid Profile and Prevalence of *spvB*-virulence Plasmids in *S. enterica* Serovars *S*. Dublin, *S*. Enteritidis, *S*. Kentucky, and *S*. Typhimurium Isolated From Domestic Animals

**Figure 2:**
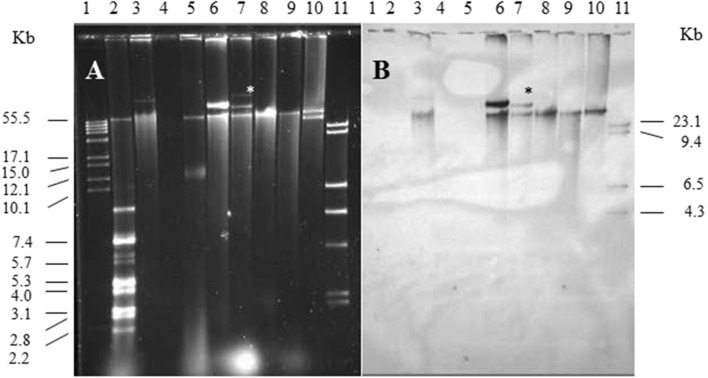
Plasmid profile and identification of *spvB*-virulence plasmid in S. Dublin and *S*. Enteritidis isolates. Gel electrophoresis of *Salmonella* plasmids **(A)**, DNA transfer, and hybridization with *spvC* DNA probe **(B)**. Lane 1: supercoiled plasmid, VI molecular weight standards (Roche); lane 2: V517 plasmid, molecular weight standards (Macrina et al., 1978); lane 3: *S*. Typhimurium LT2 (90 kb *spvB*-virulence plasmid control); lane 4: *S*. Typhimurium LT2 pSLT^−^ (*spvB*-virulence plasmid negative control); lane 5: *E. coli* XK1200 with pOX38-Km; lanes 6–8: *S*. Dublin isolates 564, 2078, and 2098; lanes 9,10: *S*. Enteritidis isolates 415 and 98; and lane 11: digoxigenin-labeled l *Hind* III molecular weight standards (Roche). *Faint, large MW plasmid that did not hybridize with *spvC* DNA probe.

**Figure 3:**
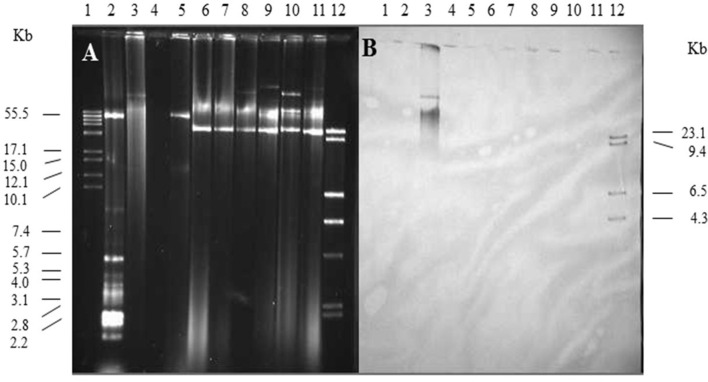
Plasmid profile and identification of *spvB*-virulence plasmid in *S*. Kentucky isolates. Gel electrophoresis of *Salmonella* plasmids **(A)**, DNA transfer, and hybridization with *spvC* DNA probe **(B)**. Lane 1: supercoiled plasmid, VI molecular weight standards (Roche); lane 2: V517 plasmid, molecular weight standards (Macrina et al., 1978); lane 3: *S*. Typhimurium LT2 (90 kb *spvB*-virulence plasmid control); lane 4: *S*. Typhimurium LT2 pSLT^−^ (*spvB*-virulence plasmid negative control); lane 5: *E. coli* XK1200 with pOX38-Km; lanes 6–11: *S*. Kentucky isolates 102, 116, 117, 112, 118, and 105; and lane 12: digoxigenin-labeled l *Hind* III molecular weight standards (Roche).

Single and multiple, large molecular weight plasmids (>55 kb) were identified in the *S. enterica* serovars screened (Figures 2, 3; and summarized in Table 4). The *spvB*-virulence plasmid was present in *Salmonella* serovars *S*. Dublin, *S*. Enteritidis, and *S*. Typhimurium, as one of these large sized plasmids (Figure 2). However, none of the *S*. Dublin *spvB*-virulence positive isolates contained the entry exclusion gene, *traS* (Table 4). Ninety-three percent of *S*. Kentucky isolates contained one or more, large molecular weight plasmids (Table 4), but none were identified as the *spvB*-virulence plasmid (Figure 3). The prevalence of other large sized plasmids (>55 kb), among *spvB-*virulence positive isolates varied from 7.7 to 62% among *S. enterica* serovars screened (Table 4). There was a statistically significant difference in the distribution of these plasmids among *S*. serovars screened relative to the prevalence of *spvB*-virulence plasmid and specifically the plasmid's resident *traS* in these same isolates (Table 4; Chi-Squared test, *p* < 0.05).

**Table 4:** Plasmid composition and prevalence of *spvB*-virulence plasmids and pSLT *traS* in *S. enterica* isolates from domestic animals.

***Salmonella* serovars^d^**	**Virulence plasmid^**a**^ (%)**	**pSLT *traS*^**b**^ (%)**	**Other^c^ plasmids ≥55 kb (%)**
*S*. Dublin (*n* = 7)	100	0	62
*S*. Enteritidis (*n* = 18)	100	100	7.7
*S*. Typhimurium (*n* =18)	100	100	24
*S*. Kentucky (*n* =14)	0	0	93
Total	75^d^	63^d^	39^d^

*Isolates were screened by PCR and DNA, DNA hybridization for spvC^a^ (virulence plasmid marker) and pSLT traS^b^*.

c *Plasmids were identified as negative for the virulence plasmid marker spvC as determined by gel electrophoresis and Southern analysis*.

d *Non-random distribution of spvB-virulence plasmid, pSLT traS or other, large molecular weight plasmids among Salmonella serovars (Chi-Squared Test: p < 0.05)*.

### Contribution of the *spvB*-virulence Plasmid and Specifically Its Resident *traS* to Exclusion of IncF Plasmids in *S*. Typhimurium

The contribution of the recipient strain's genetic background in IncF plasmid transfer, specifically focusing on *spvB*-virulence plasmid and the plasmid's resident *traS* was examined. Several *S*. Typhimurium LT2 strains were created that were negative for spvB-virulence plasmid (pSLT^−^), or contained targeted deletions in *traS* or another plasmid gene, unrelated to plasmid transfer exclusion (*spvB*). In addition to these strain constructs, *S*. Typhimurium LT2 pSLT^−^ and *E. coli* DH5α strains with the cloned FI *traS* (pRS31) (Collard et al., 2007) served as FI plasmid exclusion controls. Conjugation frequency was calculated as transconjugants per recipient (Cottell et al., 2012; Anjum et al., 2016) rather commonly used transconjugants per donor (Klimke and Frost, 1998; Ahmer et al., 1999). The latter calculation describes the plasmid's properties, in a given host, while in this circumstance, the former is preferred in describing plasmid transfer in relation to the recipient strains used in this study. The *spvB*-virulence plasmid significantly reduced FI and FII plasmid transfer 10 to 100-fold into *S*. Typhimurium LT2 recipient strain background (Table 5; *S*. Typhimurium LT2 wild-type strain vs. LT2 pSLT^−^, *p* < 0.05). Introduction of pRS31 (FI *traS*^+^) into *S*. Typhimurium LT2 pSLT^−^ strain restored plasmid exclusion of both F plasmids (Table 5; *p* < 0.05), but exclusion was most pronounced for the FI plasmid pOX38-km in *S*. Typhimurium LT2 strain [Table 5; Exclusion Index (EI) 22,741.12 vs. 83.78]. Similarly, pRS31 with FI *traS*^+^ was able to exclude F1 plasmid pOX38-km from *E. coli* DH5α and exhibited plasmid specificity in its exclusion of FI vs. FII plasmids (Table 5; Collard et al., 2007). If *spvB*-virulence plasmid's *traS* is responsible for F plasmid exclusion in *S*. Typhimurium LT2, then deletion of this gene is expected to significantly increase plasmid transfer compared to wild-type or *S*. Typhimurium LT2 strain with a deletion in another, unrelated plasmid gene. The *traS* deletion did not significantly alter plasmid transfer frequency compared to either the wild type or *spvB* deletion strain for F plasmids pOX38-km (FI) or R100 (FII) (Table 5).

**Table 5:** The contribution of the *Salmonella spvB*-virulence plasmid and the resident plasmid *traS* on exclusion of IncF plasmids.

**Plasmid**	**Recipient strain^a^**	**Plasmid transfer frequency^b^**		**EI^c^**	***P*-value^d^**
		**Average**	**SEM**		
FI (pOX38-km)	LT2R pSLT^+^	2.87 × 10^−3^	±1.26 × 10^−3^	15.61	0.034
	LT2R pSLT^−^	4.48 × 10^−2^	±1.85 × 10^−2^		
	LT2R pSLT^−^, FI *traS*^+^	1.97 × 10^−6^	±0.56 × 10^−6^	22,741.12	0.030
	LT2R pSLT^+^ Δ*traS*	7.51 × 10^−4^	±1.46 × 10^−4^	59.65	0.019, 0.104^e^
	LT2R pSLT^+^ Δ*spvB*	1.63 × 10^−3^	±0.21 × 10^−3^	27.48	0.021, 0.104^e^
	*E. coli* DH5α	7.99 × 10^−1^	±1.00 × 10^−1^		
	*E. coli* DH5α FI *traS*^+^	2.39 × 10^−3^	±1.14 × 10^−3^	334.31	<0.001^f^
FII (R100)	LT2R pSLT^+^	4.11 × 10^−5^	±2.20 × 10^−5^	301.70	0.025
	LT2R pSLT^−^	1.24 × 10^−2^	±0.48 × 10^−2^		
	LT2R pSLT^−^ FI *traS*^+^	1.48 × 10^−4^	±0.37 × 10^−4^	83.78	0.026
	LT2R pSLT^+^ Δ*traS*	5.56 × 10^−4^	±2.37 × 10^−4^	22.30	0.025, 0.044^e^
	LT2R pSLT^+^ Δ*spvB*	3.27 × 10^−5^	±1.13 × 10^−5^	379.20	0.025, 0.252^e^
	*E. coli* DH5α	2.21 × 10^−1^	±0.85 × 10^−1^		
	*E. coli* DH5α FI *traS*^+^	2.79 × 10^−1^	±0.85 × 10^−1^	0.79	0.264^f^

a *pSLT, S. Typhimurium LT2 spvB-virulence plasmid*.

b *Plasmid transfer frequency was calculated as total number of transconjugants /total number of recipients*.

c *EI (Exclusion index): plasmid transfer frequency for Salmonella (LT2) pSLT^-^ recipient/plasmid transfer frequency for S. Typhimurium recipients with: pSLT virulence plasmid, pSLT2 traS deletion, pSLT2 spvB deletion or pRS31 (FI traS^+^); plasmid transfer frequency for “plasmidless” E. coli DH5α/plasmid transfer frequency for E. coli DH5α recipient with pRS31 (FI traS^+^) (Anthony et al., 1999)*.

d *Student T-test of plasmid transfer frequencies between S. Typhimurium LT2 pSLT^-^ strain and Salmonella genotypes: pSLT^-^, ΔtraS, or ΔspvB*.

e *Student T-test of plasmid transfer frequencies between S. Typhimurium LT2 wild type strain (pSLT^+^) and Salmonella genotypes: ΔtraS, or ΔspvB*.

f *Student T-test of plasmid transfer frequencies between “plasmidless” E. coli DH5α/plasmid transfer frequency for E. coli DH5α with pRS31 (FI traS^+^)*.

### Contribution of the *spvB*-virulence Plasmid and Specifically Its Resident FII *traS* to Exclusion of IncF Plasmids in Natural *Salmonella* Isolates

The ability of *spvB*-virulence plasmid to exclude F plasmids was examined in natural *Salmonella* isolates. Critical to this study was the identification of a natural *S*. Typhimurium clone (Hudson et al., 2000; Hernandez et al., 2012), with and without the *spvB*-virulence plasmid, and several *Salmonella* isolates with the virulence plasmid, minus *traS* (Table 1). The natural isolates exhibited 20 to 700-fold, lower conjugation frequency for FI plasmid compared to the *spvB*-virulence plasmid positive, *S*. Typhimurium LT2 control (Table 6). Presence or absence of the virulence plasmid or the plasmid's *traS* in natural *Salmonella* isolates did not correlate with changes in conjugation frequency for either F plasmids (Table 6). The virulence plasmid alone does not appear to be a significant barrier to F-plasmid exclusion in these isolates.

**Table 6:** The contribution of the *Salmonella spvB*-virulence plasmid on exclusion of IncF plasmids in natural *S. enterica* isolates.

**Plasmid**	**Recipient stain^a^**	**Plasmid transfer frequency^b^**		***P*-value^c^**
		**Average**	**±SEM**	
FI (pOX38-km)	*S*. Typhimurium LT2 pSLT^+^	2.69 × 10^−3^	±1.49 × 10^−3^	0.042
	*S*. Typhimurium LT2 pSLT^−^	3.14 × 10^−2^	±1.13 × 10^−2^	
	*S*. Typhimurium 98A-28238R (*spvB*-virulence plasmid^−^)	3.42 × 10^−5^	±0.55 × 10^−5^	
	*S*. Typhimurium 98A-33516R (*spvB*-virulence plasmid^+^, pSLT *traS*^−^)	9.25 × 10^−6^	±1.21 × 10^−6^	0.034, 0.006^d^
	*S*. Dublin 564R (*spvB*-virulence plasmid^+^, pSLT *traS*^−^)	1.22 × 10^−4^	±0.56 × 10^−4^	0.035
	*S*. Choleraesuis 10708R (*spvB*-virulence plasmid^+^)	3.64 × 10^−6^	±1.46 × 10^−4^	0.035
	*E. coli* DH5α	2.23 × 10^−1^	±0.50 × 10^−1^	
FII (R100)	*S*. Typhimurium LT2 pSLT^+^	8.35 × 10^−6^	±1.38 × 10^−6^	0.049
	*S*. Typhimurium LT2 pSLT^−^	2.28 × 10^−3^	±1.28 × 10^−3^	
	*S*. Typhimurium 98A-28238R (*spvB*-virulence plasmid^−^)	1.69 × 10^−6^	±0.30 × 10^−6^	
	*S*. Typhimurium 98A-33516R (*spvB*-virulence plasmid^+^, pSLT *traS*^−^)	1.71 × 10^−5^	±0.51 × 10^−5^	0.014, 0.164^d^
	*S*. Dublin 564R (*spvB*-virulence plasmid^+^, pSLT *traS*^−^)	2.44 x10^−3^	±1.22 × 10^−3^	0.466
	*S*. Choleraesuis 10708R (*spvB*-virulence plasmid^+^)	7.85 × 10^−7^	±1.00 × 10^−7^	0.163
	*E. coli* DH5α	6.77 × 10^−2^	±4.80 × 10^−2^	

a *pSLT, S. Typhimurium LT2 spvB-virulence plasmid*.

b *Plasmid transfer frequency was calculated as total number of transconjugants /total number of recipients*.

c *Student T-test comparisons between S. Typhimurium LT2 pSLT^−^ strain and other Salmonella isolates*.

d *Student T-test comparison between spvB-virulence plasmid^−^, S. Typhimurium isolate 98A-23238R, and virulence positive isolate 98A-33516R*.

### F-plasmid Exists Autonomously in *S*. Typhimurium LT2 Containing IncF, *spvB*-virulence Plasmid

The *spvB*-virulence plasmid is comprised of the FIB and FII replicons present in F and R100 plasmids, respectively (Villa et al., 2010). These plasmid replicons play an important role in plasmid incompatibility for IncF group of plasmids (Novick, 1987). Therefore, plasmid incompatibility is expected to affect the persistence of the resident plasmid in wild-type *S*. Typhimurium LT2 (pSLT^+^) transconjugants. With continued antibiotic selection pressure on pOX38 and plasmid incompatibility, one expectation is the loss of SpvB-virulence plasmid while another possible outcome is the recombination between the two plasmids. Plasmid recombination was expected to result in change in size of either plasmid and pSLT and F-plasmid specific probes binding to the same-size DNA band(s). On the other hand, if the pSLT and F-plasmid specific probes bound to distinctly different size DNA bands, similar in size to the plasmid controls, then these plasmids existed as separate entities in *Salmonella* (pSLT^+^) transconjugants

**Figure 4:**
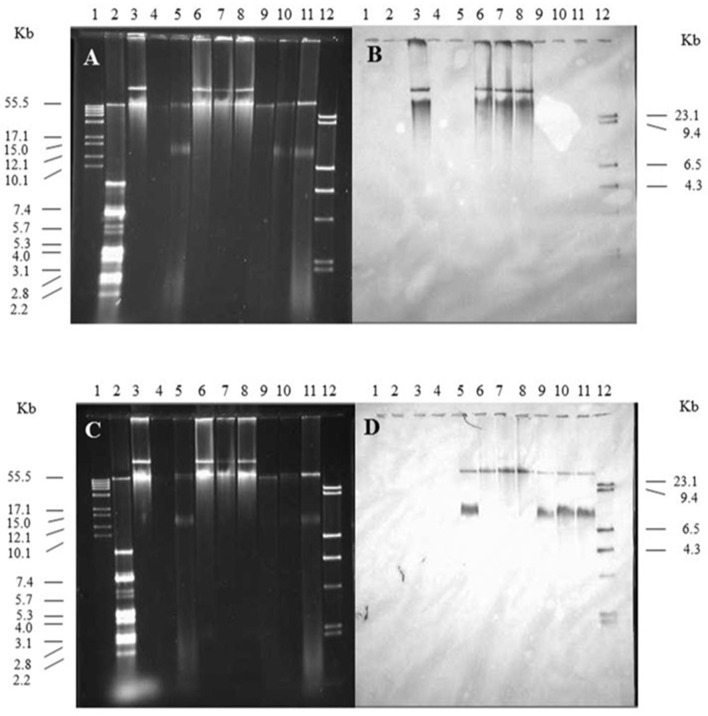
The contribution of the *spvB*-virulence plasmid on localization of F-plasmid in *S*. Typhimurium transconjugants. Gel electrophoresis of *Salmonella* plasmids **(A,C)**, DNA transfer, and hybridization with *spvC* **(B)** or FI *traS* **(D)** DNA probes. Lane 1: supercoiled plasmid, VI molecular weight standards (Roche); lane 2: V517 plasmid, molecular weight standards (Macrina et al., 1978); lane 3: *S*. Typhimurium LT2 (90 kb *spvB*-virulence plasmid control); lane 4: *S*. Typhimurium LT2 pSLT^−^ (*spvB*-virulence plasmid negative control); lane 5: *E. coli* XK1200 with pOX38-Km; lanes 6–8: *S*. Typhimurium LT2R transconjugants (pOX38-Km); lanes 9–11: *S*. Typhimurium LT2 pSLT^−^ transconjugants (pOX38-Km); and lane 12: digoxigenin-labeled l *Hind* III molecular weight standards (Roche).

Kanamycin-resistant, *S*. Typhimurium LT2 (pSLT^+^) transconjugants were positive for the SpvB-virulence plasmid. The pSLT and pOX38 controls produced two distinct, DNA bands that most likely represent their relaxed and supercoiled states (Figure 4, lanes 3 and 5, respectively). *Salmonella* Typhimurium LT2 pSLT^+^ transconjugants had similar size DNA bands, recognized by pSLT-probe as the virulence plasmid control (Figures 4A,B, lanes 6–8 vs. lane 3). However, one of the two DNA bands recognized by F-plasmid specific probe was absent in *S*. Typhimurium pSLT^+^ transconjugants (Figure 4B, lane 5 vs. lanes 6–8).

## Discussion

*traS* and *traT* are responsible for F-plasmid exclusion. In genomic comparisons of the virulence plasmid of *Salmonella* serovars, Choleraesuis, Dublin, Enteritidis, and Kentucky, the most notable genetic difference linking plasmid exclusion to the slow development of plasmid-mediated antibiotic resistance in *Salmonella* serovars was *traS*. The distribution of the *spvB*-virulence plasmid and specifically *traS* among *Salmonella* serovars adversely affects the prevalence of other large molecular weight plasmids. The virulence plasmid significantly excluded F plasmid transfer to *S*. Typhimurium LT2 strain. Introduction of pRS31 containing FI *traS* into a virulence plasmid-deficient *S*. Typhimurium LT2 restored exclusion, with plasmid specificity exhibited by *traS* (Audette et al., 2007). However, the plasmid exclusion was not linked to the plasmid's resident *traS* as its deletion did not significantly decrease exclusion compared to plasmid gene deletion control, Δ*spvB*. One possibility is the overnight mating period, even at a 1:10 donor to recipient ratio, favored multiple plasmid transfer events (Simonsen et al., 1990; Anthony et al., 1994), which overwhelmed entry exclusion by *traS* or *traT* and therefore masked the impact *traS* deletion had on plasmid exclusion. While *traS* was cloned into a low-copy number plasmid, its expression is under p*lac* promoter and in *Salmonella*, without the plac repressor *lacI*, this gene is expected to be constitutively expressed. Therefore, overexpression of TraS would explain plasmid exclusion, even under conditions conducive to multiple, repeated plasmid transfers in the recipient population, as transconjugants become new plasmid donors.

It is also possible that *traT* plays a more significant role in plasmid exclusion. While *traT* exhibits greater conservation in its amino acid sequence compared to *traS* of F and R100 plasmids (Harrison et al., 1992), like *traS* (Garcillan-Barcia and de la Cruz, 2008), *traT* exhibits plasmid specificity in its exclusion (Harrison et al., 1992). There is significant sequence divergence in F plasmid replicons and *tra* genes, including *traS* and *traT*, where F plasmid evolution in *Klebsiella, Salmonella*, and *Yersinia* mirrors the divergence of these genera (Villa et al., 2010). This in part explains how F plasmid pOX38-km can exist as an autonomous replicon in *Salmonella* host with the FII/FIC virulence plasmid pSLT but does not explain how this plasmid can exclude F plasmids from entry into the *Salmonella* cell. Maybe the pSLT TraT shares some amino acid sequence or motif with both FI and FII TraT or there is some other plasmid gene(s) responsible for F-plasmid exclusion.

While plasmid exclusion, linked to the virulence plasmid in *S*. Typhimurium laboratory strain LT2 was observed, similar plasmid exclusion was not observed for natural *Salmonella* isolates, that varied in their *spvB*-virulence plasmid or *traS* genotype. Despite conditions that favor multiple plasmid transfer events (Simonsen et al., 1990; Anthony et al., 1994), conjugation frequencies were significantly lower in natural *Salmonella* isolates compared to *S*. Typhimurium LT2 strains. As these isolates were not screened for *traT*, it is possible that while negative for *traS, traT* is sufficient for excluding IncF plasmids in these isolates. Another possibility, is that these isolates contain other IncF plasmids (Villa et al., 2010) not recognized by our *traS* probes. No difference in plasmid exclusion for two genetically related *S*. Typhimurium isolates ± *spvB* virulence plasmid was observed. In fact, the *spvB*-virulence plasmid, negative *S*. Typhimurium isolate, which one would expect to be more receptive to plasmid acquisition, acquired either IncF plasmids at a much lower frequency compared to LT2, pSLT^−^ control (10^−5^ to 10^−6^ vs. 10^−2^ to 10^−3^). Another plasmid exclusion mechanism may be involved in some *Salmonella* serovars or strains. One possible candidate for plasmid exclusion is clustered regularly interspaced short palindromic repeat (CRISPR) system that functions to exclude foreign genetic elements from entering the cell by forming a perfect sequence match between the spacer in CRISPR and the spacer located in invading DNA (Marraffini and Sontheimer, 2008; Shariat et al., 2015). Unraveling the contribution of these candidate genes (*traT*, CRISPR*-cas*) to plasmid exclusion will require creating *S*. Typhimurium strains with single or multiple deletions in targeted genes, and comparative genomics of multi-drug resistant and pan-susceptible *Salmonella* isolates.

## Conclusion

Antibiotics have been a great panacea in reducing morbidity and mortality attributed to bacterial infections. Unfortunately, resistance to these “wonder” drugs often quickly follows their introduction (Abraham and Chain, 1988). Usage of antibiotics in agriculture has long been a contentious issue; with fears that antimicrobial resistance has spilled over into human pathogens through the food chain 1975. However, there are several circumstances where there is a disconnect between antibiotic usage and resistance in bacteria colonizing food animals (Idris et al., 2006; Simjee et al., 2007; Smith et al., 2007; Liljebjelke et al., 2017). There is also a disparity in the antimicrobial susceptibility of microbes that inhabit the same environment which may be high in antibiotic resistance gene load (Nandi et al., 2004), and encounter the same selection pressures (e.g., antibiotic usage) (Simjee et al., 2007). This disparity in antimicrobial susceptibility also occurs within the same species, as is the case for *S. enterica* (Liljebjelke et al., 2017). It appears that several genetic factors are at play that affects the speed at which antibiotic resistance develops and spreads within a bacterial population. A systems-based approach will provide a better understanding of how and when antimicrobial resistance emerges in zoonotic pathogens like *Salmonella*.

## Data Availability Statement

The datasets generated for this study can be found in the NCBI BioProject PRJNA186035.

## Author Contributions

JM, ML, and EL contributed to the conception and design of this study. MO, YC, and CG were responsible for the acquisition of data analyzed in this study. JM and CG were involved in the analysis and interpretation associated with this work. All authors were involved in manuscript writing, revisions, and final approval of this manuscript.

## Conflict of Interest

The authors declare that the research was conducted in the absence of any commercial or financial relationships that could be construed as a potential conflict of interest.

## Abbreviations

Ap: ampicillin
Cm: chloramphenicol
Fa: fusaric acid
Km: kanamycin
LB: Luria-Bertani
Nal: nalidixic
PCR: polymerase chain reaction
Rif: rifampicin
Sp: spectinomycin
Sm: streptomycin
Su: sulfonamide
TAE: Tris acetate EDTA buffer
Tc: tetracycline
TSA: tryptic soy agar
TSB: tryptic soy broth
UV: ultra violet.
